# Prevalence, Risk Indicators, and Patient‐Reported Outcomes of Labial Gingival Recession Following Orthodontic Treatment: A Retrospective Study

**DOI:** 10.1002/cre2.70483

**Published:** 2026-10-11

**Authors:** Sarah Benabdallah, Parham Hazrati, Catherine Delcourt, Pierre‐Alexandre Chataigner, Nassima Ramdane, Claire Averlant‐Dubois, Hom‐Lay Wang, Kevimy Agossa

**Affiliations:** ^1^ Department of Orthodontics University of Lille, Lille University Hospital Lille France; ^2^ Department of Oral Medicine, Infection, and Immunity Harvard School of Dental Medicine Boston Massachusetts USA; ^3^ Private Practice Bondues France; ^4^ Private Practice Tournai Belgium; ^5^ Department of Biostatistics CHU Lille Lille France; ^6^ Department of Periodontics and Oral Medicine University of Michigan School of Dentistry Ann Arbor Michigan USA; ^7^ Department of Periodontology School of Dentistry, University of Lille, CHU Lille, INSERM U1008, Advanced Drug Delivery Systems Lille France

**Keywords:** gingival recession, orthodontic treatment, patient‐reported outcome, periodontal phenotype, retrospective study

## Abstract

**Objectives:**

To assess the prevalence, distribution, and risk indicators of gingival recession (GR) following orthodontic treatment (OT) in both private and hospital‐based settings, and to evaluate its impact on patient satisfaction and willingness to seek corrective treatment.

**Materials and Methods:**

Clinical and radiographic records from baseline to follow‐up were retrospectively analyzed. Additionally, participants completed a survey on satisfaction, perception of GR if present, and willingness to seek corrective treatment. Incident and persistent GR were analyzed separately. Bayesian mixed‐effects logistic regression with weakly informative priors was used to identify risk indicators for incident GR.

**Results:**

A total of 103 patients (2030 teeth; mean age: 20.7 ± 9.7 years) were included. GR prevalence increased from 1.7% at baseline to 2.0% at appliance removal and continued to rise during the retention phase (3%–5%), with a site‐specific distribution. Among 1996 teeth free of GR at baseline, 49 developed incident GR. A thin‐scalloped gingival phenotype was the strongest risk indicator for incident GR (OR = 4.10; 95% CrI: 1.25–13.46). Most baseline GR persisted throughout follow‐up. Satisfaction with OT remained high (mean score: 8.99 ± 1.04 on a 10‐point scale) and was not significantly influenced by GR or treatment setting.

**Conclusions:**

Gingival recession occurrence after orthodontic treatment is time‐ and site‐dependent. A thin‐scalloped gingival phenotype is the main risk indicator for incident GR, whereas pre‐existing recession is highly persistent. Despite its occurrence, gingival recession had limited impact on patient satisfaction.

**Clinical Relevance:**

This study provides long‐term, multicenter data on gingival recession following orthodontic treatment, and is the first to assess its impact on patient perception. The findings may help inform risk‐based treatment planning and individualized patient counseling in orthodontic care.

## 1 Introduction

Orthodontic treatment (OT) involves complex biomechanical interactions between teeth and surrounding periodontal structures, which can result in transient or permanent periodontal alterations (Li et al. 2021; Martin et al. 2022; Papageorgiou et al. 2018). One of the most clinically relevant adverse outcomes is labial gingival recession (GR), which may arise or progress during active OT or in the post‐treatment retention phase (Wennström 1996; Jepsen et al. 2023). Several studies have reported a higher prevalence of GR among orthodontically treated individuals compared to untreated controls (Allais 2003; Slutzkey and Levin 2008; Juloski et al. 2017; Renkema et al. 2013a). Longitudinal data further suggest that GR prevalence increases over time following treatment completion (Renkema et al. 2013b; Gebistorf et al. 2018)

Existing evidence on risk factors for GR in orthodontically treated patients remains inconsistent and heterogeneous (Joss‐Vassalli et al. 2010). While certain studies implicate specific tooth movements—such as labial proclination and transverse expansion—as contributing to GR (Allais 2003; Slutzkey and Levin 2008; Juloski et al. 2017), others emphasize individual susceptibility factors, including thin gingival phenotype or alveolar bone morphology (Yared et al. 2006; Mandelli et al. 2025). A recent review highlighted significant methodological limitations that may account for these discrepancies, including absence of post‐treatment cephalometric data, lack of control groups, and inadequate adjustment for confounding variables such as oral hygiene or baseline gingival conditions (de Llano‐Pérula et al. 2023).

With the growing demand for OT, especially among adult patients, the esthetic and functional consequences of GR have gained prominence as a clinically significant concern (Jepsen et al. 2023; Saloux et al. 2022). Periodontal complications arising during or after OT are among the leading causes of litigation in orthodontics, underscoring their clinical and medico‐legal importance (Kokich 2013). Despite this, no study to date has comprehensively assessed the perceived impact of GR from the patient's perspective, including its influence on satisfaction with OT outcomes or the request of corrective procedures. Better insight into orthodontic patients’ perceptions of GR is crucial for advancing individualized care and long‐term preventive strategies.

The present study aimed to: (a) assess the prevalence and anatomical distribution of GR in patients followed up to 10 years after OT in both private and hospital‐based settings; (b) identify risk indicators associated with GR occurrence; and (c) assess the impact of GR on patient‐reported satisfaction and requests for corrective treatment. We tested two null hypotheses: (1) there is no significant variation in GR prevalence over time or between treatment settings; and (2) GR does not significantly affect patient satisfaction with OT.

## 2 Methods

### 2.1 Study Design and Setting

This multicenter retrospective cohort study with cross‐sectional assessment of patient satisfaction included patients treated at the orthodontic department of Lille University Hospital (France) and two private orthodontic practices in Lille (France) and Tournai (Belgium). It involved the analysis of clinical and radiographic data collected during routine care, as well as patient responses to a routine post‐treatment satisfaction questionnaire. The study adhered to STROBE guidelines (von Elm et al. 2008).

### 2.2 Ethical Approval

Under the applicable French and Belgian regulations, studies based solely on retrospective analysis of existing data and satisfaction surveys are exempt from ethics committee review but must comply with the European General Data Protection Regulation (GDPR) requirements. Accordingly, the study protocol was reviewed and approved by the Data Protection Officer (DPO) Committee of Lille University Hospital (approval no. 2023‐157), a regulatory body that oversees GDPR compliance for research involving personal health data. The Belgian participating center contributed anonymized data under the same European data‐protection (GDPR) requirements. All participants provided informed consent. The study was conducted in line with the Declaration of Helsinki (1975, revised 2013).

### 2.3 Participants and Eligibility Criteria

Eligible participants had completed fixed appliance OT and had high‐quality inter‐arch photographs at baseline (T0), at appliance removal (T1), and at least one of the following retention time points:

- T2: ≤ 6 months,
- T3: > 6 and < 12 months,
- T4: ≥ 12 and < 24 months,
- T5: ≥ 24 months post‐treatment.

Patients were recruited from both hospital and private settings to explore potential center‐related outcome variations. Private practices were selected on a voluntary basis from regular referring orthodontists.

Exclusion criteria:

- Syndromic patients or those with clefts/craniofacial anomalies
- Absence of fixed mandibular anterior retainers

No participant had undergone gingival grafting before OT.

### 2.4 Outcomes and Data Collection

Primary outcome: Tooth‐level presence of GR, defined as visible root exposure apical to the cementoenamel junction (CEJ) (Cortellini and Bissada 2018), assessed from intraoral photographs (Figure 1). GR at baseline was confirmed from patient records and photographs. Molars were excluded due to visibility limitations. Photographs were taken in occlusion from three angles (frontal, right, left) and independently evaluated by two calibrated examiners (K.A. and S.B.). Discrepancies were resolved by consensus.

**Figure 1:**
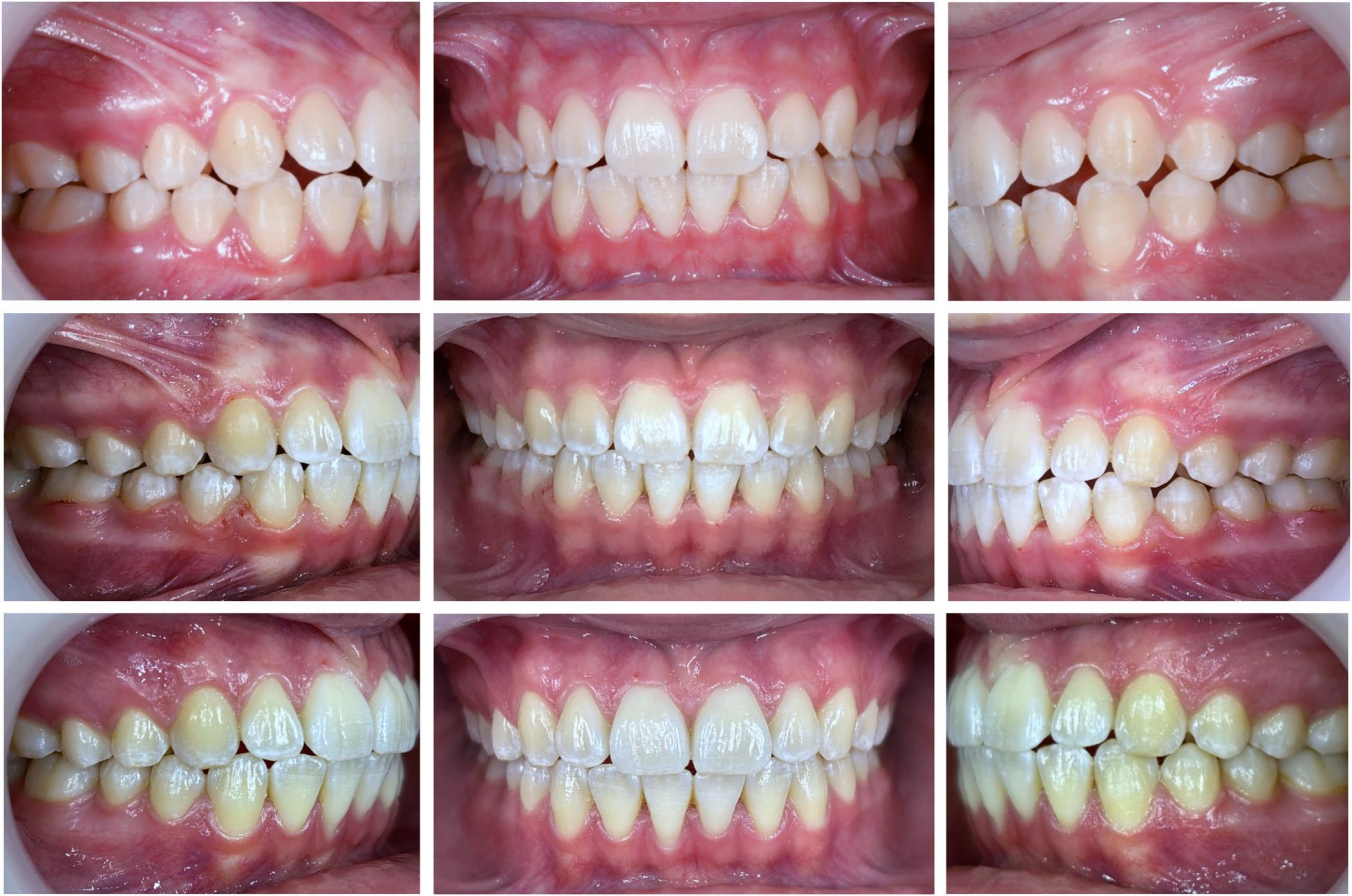
Representative intra‐arch photographs of a patient before orthodontic treatment (OT), at the end of OT, and 3 years after appliance removal. Note the development of gingival recession (GR) on tooth 41 during the retention phase.

Secondary outcomes: Patient‐reported outcomes (PROs), assessed via a structured satisfaction questionnaire during retention follow‐up.

### 2.5 Variables Collected

Patient‐level variables:

- Age at follow‐up (years)
- Gender (M/F)
- Socio‐professional category
- Treatment setting: hospital (HOSP) vs. private (PRIV)
- Duration of active treatment (months)
- Duration of retention (months)
- Smoking status: current, former, e‐cigarette user, with consumption history

Mouth‐level variables:

- Angle classification
- Overjet (mm)
- Overbite (%)
- Crossbite (tooth number)
- Treatment modalities: arch expansion, extractions, or orthognathic surgery

Tooth‐level variables:

- Presence of GR (Y/N); severity at baseline (≤ 1 mm, > 1 mm)
- Gingival phenotype classified according to the Seibert and Lindhe system (Seibert and Lindhe 1989; Dziewulska et al. 2025):

– Thin‐scalloped: Narrow zone of keratinized tissue (< 2 mm) with clear, thin, delicate gingiva.
– Thick‐flat: Broad zone of keratinized tissue (≥ 2 mm) with dense, fibrotic gingiva.

### 2.6 Radiographic Measurements

Cephalometric measurements were performed on lateral cephalograms using Fiji/ImageJ (v1.54 f, NIH, USA) (Schindelin et al. 2012; Rueden et al. 2017). The following variables were recorded:

- ANB angle (°): maxillo‐mandibular skeletal discrepancy
- FMA angle (°): mandibular plane inclination
- I/F angle (°): upper incisor inclination to Frankfort plane
- IMPA (°): lower incisor inclination to mandibular plane
- W (mm): mandibular symphysis width
- H (mm): symphysis height

All measurements were performed by a trained examiner. Intra‐rater reliability was assessed using intra‐class correlation coefficients (ICC) on a second set of 15 randomly selected radiographs, remeasured ≥ 3 months later under the same conditions.

### 2.7 Patient‐Reported Outcome Measures (PROMs)

A custom questionnaire was designed based on the literature (Suryanti et al. 2020; Elkerbout et al. 2023; Santiwong et al. 2022; Salvesen et al. 2022). It included:

1. Oral hygiene behavior, based on OHB questionnaire (Suryanti et al. 2020; Elkerbout et al. 2023): brushing frequency, technique, bleeding, duration, and tool type (see Supporting Information S1)
2. Brushing force, assessed via 0–10 Visual Analog Scale (VAS); a composite traumatic brushing score was computed using force, duration, bristle hardness, and technique (see Supporting Information S1)
3. Orthodontic treatment satisfaction, using a 6‐point Likert scale (alignment, smile/facial aesthetics, function, self‐esteem) and a global satisfaction VAS (0–10)
4. Perception of GR, assessed with a 0–10 VAS for both esthetic impact and desire for corrective treatment (in patients with GR).

### 2.8 Statistical Analysis

Descriptive analyses summarized sociodemographic, orthodontic, and periodontal variables using means ± SD for continuous data and frequencies for categorical data. Likert items were visualized using stacked bar charts.

Data normality was assessed with the Shapiro–Wilk test. Intergroup differences were tested with the Kruskal–Wallis test for non‐parametric data, followed by Wilcoxon rank‐sum tests with continuity correction. Chi‐squared tests were used for categorical variables.

The primary outcome was incident GR, defined as the development of new root exposure at teeth without GR at baseline. This definition was adopted to distinguish newly occurring GR after OT from pre‐existing GR that persisted throughout follow‐up. Because incident GR events were infrequent, associations between potential risk indicators and incident GR were evaluated using Bayesian mixed‐effects logistic regression with weakly informative Student‐t priors (location = 0, scale = 2.5), allowing stable estimation in the presence of sparse data. Tooth‐level observations were nested within patients using a random intercept to account for intra‐patient clustering. The prespecified primary model included age, sex, gingival phenotype, baseline ANB angle, baseline IMPA, and follow‐up duration as fixed effects. Effect estimates were reported as odds ratios (ORs) with 95% credible intervals (CrIs), reflecting posterior uncertainty. Posterior probabilities of the direction of association (Ppos) were additionally calculated to quantify the probability that the estimated effect was in the observed direction.

Variables assessed after the occurrence of GR, including patient‐reported perception of recession, dentin hypersensitivity, and aesthetic concerns, were considered potential consequences of GR rather than baseline risk indicators and were therefore excluded from the regression model. Persistence of baseline GR was summarized descriptively.

All analyses were conducted by two authors with experience in biostatistics (P.H. and N.R.) using RStudio (v2026.06.0 + 242) with packages dplyr, tidyr, forcats, patchwork, brms, and logistf (Bürkner 2017; Heinze and Schemper 2002).

## 3 Results

### 3.1 Patient and Site Characteristics

A total of 2030 teeth from 103 patients (34 males and 69 females; sex ratio 1:2.0) were included in the study. The mean age of participants was 20.7 ± 9.7 years. Most were non‐smokers (90%). Forty‐eight patients were treated in a hospital setting (HOSP), while 55 were treated in private practice (PRIV). A detailed participant flowchart is presented in Figure 2, and patient/site characteristics are summarized in Table 1. Clinical photographs during the retention phase were available for 31 patients at T2 (≤ 6 months), 32 at T3 (> 6 and < 12 months), 37 at T4 (≥ 12 and < 24 months), and 16 at T5 (≥ 24 months post‐treatment). Lateral cephalograms before and after treatment were available for 37 patients.

**Figure 2:**
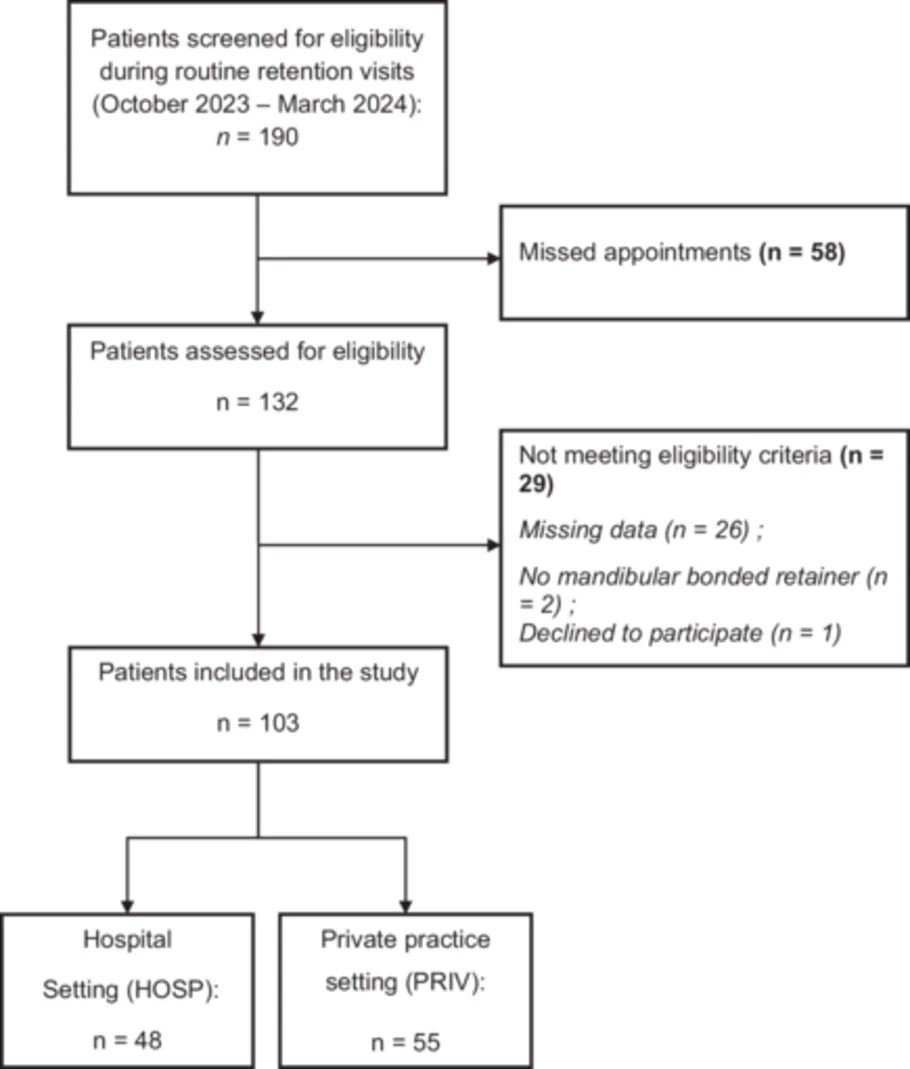
Flowchart of patient selection process.

**Table 1:** Prevalence of gingival recession at the patient‐ and tooth‐level at baseline, after orthodontic treatment, and during follow‐up, stratified by treatment setting (HOSP, PRIV, combined).

Center	Level	Baseline	After active OT^a^	Less than 6 months of retention	6 to 12 months of retention	12 to 24 months of retention	More than 24 months of retention
HOSP^b^	Patient	16.7%	16.7%	14.6%	2.1%	6.2%	2.1%
	Tooth	2.4%	1.9%	3.2%	2%	5.1%	12.5%
PRIV^c^	Patient	3.6%	10.9%	0%	10.9%	10.9%	5.5%
	Tooth	1.1%	2%	0%	3.4%	3.5%	3.9%
Total	Patient	9.7%	13.6%	6.8%	6.8%	8.7%	3.9%
	Tooth	1.7%	2%	3%	3%	3.8%	5%

^a^ OT: Orthodontic treatment.

^b^ Hospital‐based setting.

^c^ Private practice.

Bivariate analysis revealed several differences between HOSP and PRIV groups, including average treatment duration (HOSP: 22.9 ± 7.8 months; PRIV: 27.7 ± 9.8 months, *p* < 0.01), retention phase duration (HOSP: 7.5 ± 5.5 months; PRIV: 20.9 ± 19.6 months, *p* < 0.001), socio‐professional profile, and certain occlusal and cephalometric parameters (see Supporting Information S2).

### 3.2 Prevalence, Incidence, and Persistence of Gingival Recession

At baseline (T0), labial GR was observed in 9.7% of patients (1.7% of teeth). At the time of appliance removal (T1), the prevalence increased to 13.6% of patients (2.0% of teeth) (Table 1). Among patients with GR at T1, 57.1% had existing GR at baseline.

During the retention phase, 49 new GR were identified in 20 patients. The most frequently affected teeth across all time points were the mandibular central incisors and first premolars, followed by the maxillary canines and first premolars (Figure 3; Supporting Information S3).

**Figure 3:**
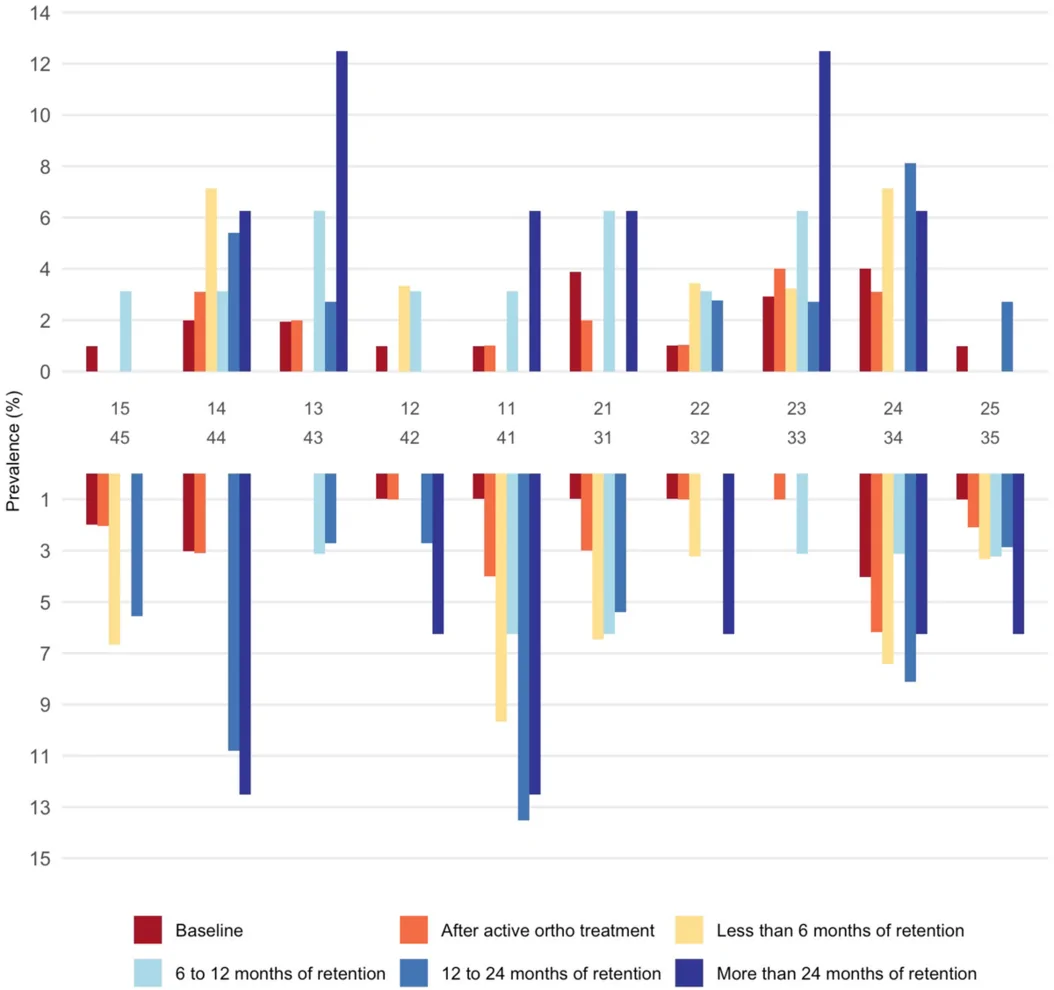
Histogram showing the anatomical distribution of gingival recession by tooth at baseline, after orthodontic treatment, and during follow‐up time points.

Among the 1996 teeth without GR at baseline, 49 developed incident GR during follow‐up, corresponding to 69 tooth‐level events across 20 patients. The proportion of teeth developing new recession increased over time, from 0.8% at appliance removal to 3.2% at ≥ 24 months of retention. Persistence of baseline GR was high throughout follow‐up: 23 of 34 affected teeth (67.6%) remained affected at appliance removal.

### 3.3 Oral Hygiene and Gingival Health

Self‐reported oral hygiene behavior and gingival health during the retention phase were assessed through a structured questionnaire completed by 99 of 103 patients (response rate: 96%). Approximately half of the respondents (51/99) reported suboptimal oral hygiene practices (inadequate brushing frequency/duration or absence of interdental cleaning). Gingival bleeding, either occasional or frequent, was reported by 22% of patients. Toothbrushing was assessed as traumatic in 64 of the 99 respondents (65%) (see Supporting Information S2).

### 3.4 Risk and Protective Indicators of GR

Risk indicator analyses were restricted to teeth free of GR at baseline (*n* = 1996), among which 69 incident GR events were recorded during retention (16, 14, 11, 18, and 10 events at appliance removal, ≤ 6 months, > 6–12 months, ≥ 12–24 months, and ≥ 24 months, respectively; Tables 1 and 2).

**Table 2:** Incidence, persistence, and prevalence of recession defects throughout the study follow‐up period.

Time point	Incident GR, *n* (%)^a^	Persistent baseline GR, *n* (%)^a^	Prevalence of GR, *n* (%)^a^
Baseline	—		34 (1.7)
After active OT^b^	16 (0.8)	23 (67.6)	39 (2.0)
≤ 6 months of retention	14 (2.4)	4 (30.8)	18 (3.0)
> 6 to < 12 months of retention	11 (1.7)	8 (80.0)	19 (3.0)
≥ 12 to < 24 months of retention	18 (2.5)	10 (90.9)	28 (3.8)
≥ 24 months of retention	10 (3.2)	6 (75.0)	16 (5.0)

^a^ Percentages are calculated among teeth with available data at each time point.

^b^ OT: Orthodontic treatment.

In the prespecified Bayesian mixed‐effects logistic regression model, a thin gingival phenotype was associated with increased odds of incident GR (OR = 4.10; 95% CrI: 1.25–13.46; Ppos = 0.99; Table 3). Older age at baseline showed an uncertain positive association with incident GR (OR = 1.11 per year; 95% CrI: 0.79–1.55; Ppos = 0.73). Male sex showed a negative but uncertain association (OR = 0.72; 95% CrI: 0.41–1.28; Ppos = 0.13). Similarly, baseline orthodontic variables (ANB and IMPA) showed no clear evidence of an association with incident GR (ANB: OR = 0.71; 95% CrI: 0.33–1.52; Ppos = 0.19; IMPA: OR = 0.94; 95% CrI: 0.85–1.04; Ppos = 0.11).

**Table 3:** Bayesian mixed‐effects logistic regression model for incident gingival recession during retention.

New recession		Bayesian mixed‐effects logistic model			
Variable	Value	Odds ratio	Lower CrI^a^	Upper CrI^a^	P‐pos^b^
Age	Years	1.11	0.79	1.55	0.73
Sex (vs. female)	Male	0.72	0.41	1.28	0.13
Baseline ANB^c^	Angles	0.71	0.33	1.52	0.19
Baseline IMPA^d^	Angles	0.94	0.85	1.04	0.11
Phenotype (vs. thick)	Thin	4.10	1.25	13.46	0.99
Follow‐up (vs. after appliance removal)	After active orthodontic treatment	2.02	0.45	9.10	0.82
	Less than 6 months of retention	8.21	0.92	73.65	0.97
	6 to 12 months of retention	2.32	0.08	64.59	0.68
	12 to 24 months of retention	6.20	0.33	117.35	0.90
	More than 24 months of retention	11.47	1.07	123.21	0.98

^a^ CrI: Credible interval.

^b^ P‐pos: Posterior probabilities of the direction of association.

^c^ ANB: Angle corresponding to maxillo‐mandibular skeletal discrepancy.

^d^ IMPA: Angle corresponding to lower incisor inclination to mandibular plane.

The odds of incident GR increased with longer follow‐up duration, with the highest estimate observed beyond 24 months after appliance removal (OR = 11.47; 95% CrI: 1.07–123.21; Ppos = 0.98). A similarly increased association was observed during the first 6 months of retention (OR = 8.21; 95% CrI: 0.92–73.65; Ppos = 0.97), whereas intermediate follow‐up intervals showed limited evidence of association (Table 3).

Substantial clustering was observed at both the patient and tooth levels (random‐intercept SD = 4.52 and 4.56, respectively), indicating that incident GR was concentrated within a subset of patients and teeth.

### 3.5 Patient‐Reported Outcomes

#### 3.5.1 Overall Satisfaction

Patients reported high levels of overall satisfaction with their OT, with a mean VAS score of 8.99 ± 1.04 out of 10. Satisfaction was significantly higher among female patients (9.18 ± 1.00) compared to males (8.61 ± 1.03; *p* = 0.0039). No significant differences were found between patients treated in HOSP and PRIV settings. The most positively rated aspects of OT were dental alignment (mean score: 5.83 ± 0.50 on a 0–6 scale), smile aesthetics (5.52 ± 0.66), and self‐esteem (5.02 ± 1.29) (Figure 4; Supporting Information S4).

**Figure 4:**
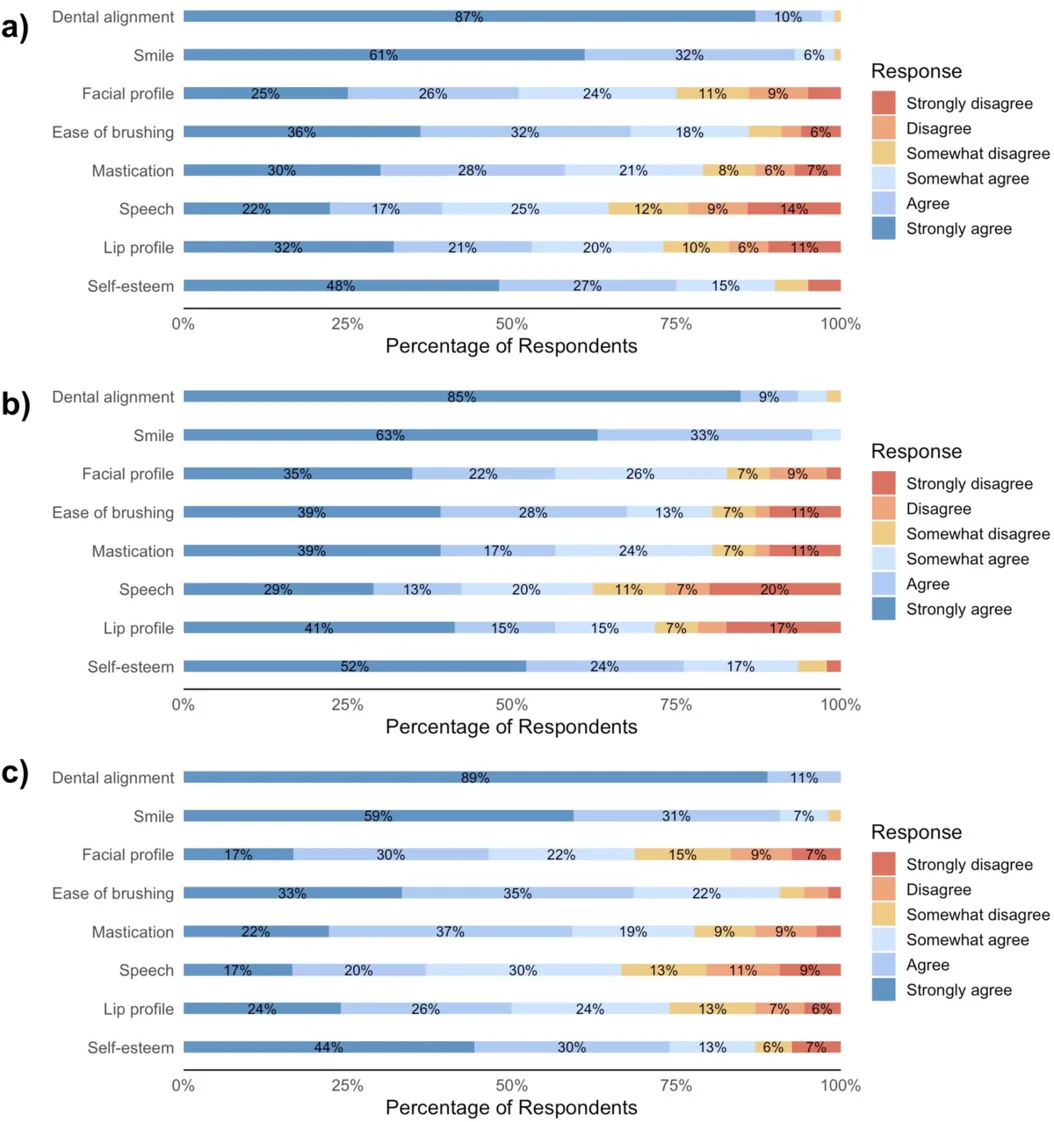
Stacked histograms representing patient satisfaction with orthodontic treatment outcomes for each item: (a) Combined HOSP + PRIV group, (b) HOSP group only, and (c) PRIV group only.

#### 3.5.2 Perception of GR and Treatment Requests

Among the 25 patients who developed GR between T0 and T5, 23 completed the relevant questionnaire section. Of these, 10 were aware of their GR, with 6 reporting that they had been informed by their dentist. Six patients reported dentin hypersensitivity, and two cited esthetic concerns. The mean VAS score for intention to seek corrective treatment was 6.6 ± 3.4 (scale: 0–10). Notably, satisfaction scores did not differ significantly between patients who developed GR and those who did not (9.10 ± 0.83 vs. 8.96 ± 1.09; *p* = 0.861) (Table 4).

**Table 4:** Patient satisfaction with overall treatment outcomes: Subgroup comparisons.

Associated factors		Overall satisfaction	*p* value
Center	HOSP^a^	9.02 ± 1.02	0.76
	PRIV^b^	8.96 ± 1.06	
Gender	Male	8.61 ± 1.03	0.0039
	Female	9.18 ± 1.00	
Age	Years	8.99 ± 1.04	0.9648
Presence of GR^c^	Yes	9.10 ± 0.83	0.8615
	No	8.96 ± 1.09	
Severity of GR	< 1 mm	9.22 ± 0.67	0.7006
	> 1 mm	9.00 ± 0.95	
GR noticed by the patient	Yes	8.82 ± 1.47	0.9902
	No	8.95 ± 1.03	
	No judgment	9.04 ± 0.88	
Dentinal hypersensitivity	Yes	8.83 ± 1.59	0.7265
	No	8.94 ± 0.97	

^a^ HOSP: Hospital setting.

^b^ PRIV: Private setting.

^c^ GR: Gingival recession.

## 4 Discussion

### 4.1 Prevalence and Distribution of GR

This multicenter study investigated the prevalence, risk indicators, and PROs related to labial GR in individuals followed for up to 111 months after OT in both HOSP and PRIV settings. The prevalence of GR increased from 9.7% of patients (1.7% of teeth) at baseline to 13.6% (2.0% of teeth) at appliance removal. Incident GR continued to develop during the retention phase, reaching 3%–5% of teeth after 6 to > 24 months of follow‐up. Mandibular central incisors and premolars were the most susceptible to GR, followed by maxillary canines and first premolars—sites typically exhibiting thin gingival phenotype and/or buccal bone dehiscence (Joshipura et al. 1994; Dridi et al. 2024).

While our findings confirm the time‐dependent and site‐specific nature of GR development in orthodontic patients, prevalence estimates in the literature vary widely—from 4.7% to 55.7% at the patient level and 5.8% to 41.7% at the tooth level (Renkema et al. 2013a, 2013b; Gebistorf et al. 2018; Mandelli et al. 2025; Vasconcelos et al. 2012). The comparatively lower prevalence observed in our cohort may be attributed to the younger age of participants (mean: 20.7 ± 9.7 years) and attrition bias, with only 16 of 103 participants completing follow‐up beyond 24 months. Because GR is also age‐related in the general population, establishing the specific contribution of OT to GR over extended time frames remains challenging (Joss‐Vassalli et al. 2010). A recent longitudinal study reported the peak of incidence of GR between appliance removal and 1 year post‐treatment (Mandelli et al. 2025) which may reflect the resolution of plaque‐induced inflammation, post‐treatment soft tissue remodeling, and age‐related skeletal growth contributing to an apical shift of the gingival margin (Verrusio et al. 2018; Guo et al. 2017).

### 4.2 Risk Indicators for Incident GR

A thin‐scalloped gingival phenotype emerged as the strongest risk indicator for incident GR among teeth free of recession at baseline (OR = 4.10; 95% CrI: 1.25–13.46; p‐positive = 0.99).

This finding is consistent with prior reports linking thin phenotype to increased susceptibility to recession, including in orthodontically treated populations (Yared et al. 2006; Mandelli et al. 2025). Findings from another study have also suggested a combined effect of gingival phenotype and mandibular incisor proclination: in mandibular incisors with a thin phenotype and proclination exceeding 95°, up to 75% exhibited GR, compared to fewer than 4% in those with a thicker phenotype (Yared et al. 2006). Such interactions may partly explain the heterogeneity in reported risk factors across studies and warrant systematic investigation in future research.

A time‐dependent increase in GR incidence was observed, with the highest odds at > 24 months of retention (OR = 11.47; 95% CrI: 1.07–123.21; p‐positive = 0.98). However, the wide credible interval reflects substantial uncertainty related to the limited number of events at this time point, and the magnitude of this association should therefore be interpreted cautiously.

Demographic and cephalometric characteristics (age, sex, baseline ANB, and baseline IMPA) showed greater uncertainty in their associations with incident GR, after accounting for gingival phenotype and follow‐up duration, with 95% CrIs spanning the null value and low Ppos values.

### 4.3 Patient‐Reported Outcomes

To our knowledge, this is the first study to evaluate how post‐orthodontic GR influences patient satisfaction and the intention to seek corrective treatment. Overall satisfaction with OT remained high (median score: 9/10; interquartile range: 9–10), consistent with previous reports (Salvesen et al. 2022). Satisfaction levels did not significantly differ between patients with and without GR (9.10 ± 0.83 vs. 8.96 ± 1.09; *p* = 0.861), nor between HOSP and PRIV settings. Interestingly, female patients reported higher satisfaction levels, independent of age.

Most GR cases were not perceived by patients. Only 10 of the 25 individuals who developed GR were aware of their condition, and of these, six reported being informed by their dentist. The average intention to request corrective treatment was moderate (mean score: 6.6 ± 3.4 on a 0–10 scale), although the extent to which this intention was influenced by professional advice remains uncertain. For comparison, in the general population, approximately 75% of GR cases go unnoticed, and only about one in 10 individuals who perceive recession actively seek treatment (Nieri et al. 2013).

### 4.4 Strengths and Limitations

This study's strengths include its multicenter design, inclusion of both hospital and private practice settings, extended follow‐up intervals, and integration of both clinical and patient‐reported outcomes. However, several limitations must be acknowledged.

Inherently, the retrospective design precludes causal inference and limited the availability and standardization of some clinical data, despite efforts to improve consistency through double photographic assessment and cephalometric analyses performed by a calibrated orthodontist. Photographic assessment of GR may introduce misclassification, particularly for shallow recessions or areas with marginal inflammation. However, such bias is expected to be non‐differential across subgroups and timepoints.

Gingival phenotype was assessed using a pragmatic visual classification based on the Seibert and Lindhe system. Although practical for retrospective orthodontic records, this approach remains subjective and less precise than instrumental methods, including ultrasonography (Dziewulska et al. 2025, 2021; Nisanci Yilmaz et al. 2022; Uysal et al. 2025; Sabri et al. 2025; Kong et al. 2023; Rodrigues et al. 2023; Malpartida‐Carrillo et al. 2021). Nevertheless, visual assessment has shown moderate‐to‐high agreement with direct measurements among experienced clinicians (Dziewulska et al. 2025).

Attrition was substantial, with long‐term follow‐up available for only one‐third of the original cohort. Missing data reduced statistical power and limited subgroup analyses. However, the consistency of our findings with previous studies in comparable populations provides some support for the robustness of the main conclusions (Gebistorf et al. 2018; de Llano‐Pérula et al. 2023).

Important potential confounders, particularly gingival inflammation and traumatic toothbrushing, were not prospectively monitored. Although these factors were partially explored through post‐treatment questionnaires, future longitudinal studies should incorporate standardized assessment throughout orthodontic treatment.

GR was an infrequent outcome, with only 69 incident events available for modeling. Consequently, only a limited number of predictors could be included in the model. Although Bayesian mixed‐effects modeling improved estimation under sparse data, uncertainty remained substantial, as reflected by the wide credible intervals.

This study focused on labial surfaces of non‐molar teeth. Lingual recession, particularly in mandibular anterior teeth, as well as “black triangles,” which are also clinically relevant following OT (Jepsen et al. 2023), were not assessed and should be addressed in future investigations.

Finally, generalizability may be limited. Participating orthodontists were voluntarily recruited from an existing referral network, which may have introduced selection bias toward clinicians with greater awareness of periodontal complications and potentially more conservative treatment approaches. Larger prospective studies are needed to better characterize the risk indicators for incident GR and to further investigate its patient‐reported impact following OT.

## 5 Conclusion

Within the limitations of this study, the following conclusions were drawn:

i. Gingival recession (GR) in orthodontic patients exhibits a time‐dependent and site‐specific pattern, with the proportion of affected teeth increasing from 1.7% at baseline to 2.0% at appliance removal and continuing to rise during the retention phase (3%–5%). Mandibular central incisors and premolars were the most frequently affected, followed by maxillary canines and first premolars.
ii. A thin‐scalloped gingival phenotype was identified as a risk indicator for incident GR (OR = 4.10; posterior probability = 0.99). Baseline recession showed high persistence throughout follow‐up (67.6%–90.9%).
iii. Overall satisfaction with orthodontic treatment was high and was not significantly influenced by the presence of GR or by treatment setting (hospital vs. private practice). Most GR cases were not perceived by patients.

## Author Contributions

**Sarah Benabdallah:** conceptualization (supporting), study design (supporting), data collection (lead), data interpretation (equal), writing – review and editing (equal). **Parham Hazrati:** data analysis (lead), data interpretation (supporting), writing – review and editing (equal). **Catherine Delcourt:** data collection (supporting), writing – review and editing (equal), validation (equal). **Pierre‐Alexandre Chataigner:** data collection (supporting), writing – review and editing (equal), validation (equal). **Nassima Ramdane:** data analysis (supporting), data interpretation (supporting), writing – review and editing (equal). **Claire Averlant‐Dubois:** data interpretation (supporting), writing – review and editing (equal), supervision (supporting). **Hom‐Lay Wang:** writing – review and editing (equal), supervision (supporting), validation (lead). **Kevimy Agossa:** conceptualization (lead), study design (lead), writing – review and editing (equal), data interpretation (equal), writing – original draft preparation (lead).

## Consent

All participants provided informed consent.

## Conflicts of Interest

The authors declare no conflicts of interest.

## Supporting information


**Supplemental Material 1**. Calculation method for oral hygiene and traumatic tooth brushing scores.
**Supplemental Material 2**. Demographic and clinical characteristics of the study population.
**Supplemental Material 3**. Tooth‐level prevalence of gingival recession at each time point.
**Supplemental Material 4**. Detailed item‐by‐item satisfaction scores by treatment setting (HOSP and PRIV).

## Data Availability

The data that support the findings of this study are available on request from the corresponding author. The data are not publicly available due to privacy or ethical restrictions.
