# Supplementary material for: Prevalence, Risk Indicators, and Patient‐Reported Outcomes of Labial Gingival Recession Following Orthodontic Treatment: A Retrospective Study
Source: Clin Exp Dent Res. 2026 Oct 11;12(5):e70483. doi: 10.1002/cre2.70483 (PMC13654315; doi:10.1002/cre2.70483)
Supplement: Supplementary file 1 [file CRE2-12-e70483-s001.docx]

**Supplemental material 1:**

|  | **Good oral hygiene** | | **Poor oral hygiene** | |
| --- | --- | --- | --- | --- |
|  | *Items* | *Score* | *Items* | *Score* |
| Frequency of brushing | 2 times per day | 2 | Not every day | 0 |
|  | >2 times per day | 3 | Once a day | 1 |
| Duration | 2 to 3 minutes | 2 | <1minute | 0 |
|  | >3 minutes | 3 | 1 to 2 minutes | 1 |
| Interdental brushing | Occasionally | 2 | Never | 1 |
|  | ≥1 time per day | 3 | Occasionally | 2 |
| Total Score | 6 to 9 | | 1 to 5 | |

|  | **Traumatic tooth brushing** | | **Minimally traumatic brushing** | |
| --- | --- | --- | --- | --- |
|  | *Items* | *Score* | *Items* | *Score* |
| Duration | >3 minutes | 3 | 2 to 3 minutes  1 to 2 minutes  <1 minute | 2  1  0 |
|  | 2 to 3 minutes | 2 |  |  |
|  | 1 to 2 minutes | 1 |  |  |
|  | <1 minute | 0 |  |  |
| Applied force (VAS) | VAS | 5 to 10 | VAS | <5 |
| Bristle hardness | Hard | 3 | Soft | 1 |
|  | Medium | 2 |  |  |
| Brushing technique | Horizontal | 3 | Vertical | 2 |
|  |  |  | Circular | 1 |
| Total Score | 10 to 19 | | 1 to 9 | |

**Supplemental material 2:**

|  | |  | | **Hospital (n = 48)** | **Private (n = 55)** | | **n** | | **P value** | |
| --- | --- | --- | --- | --- | --- | --- | --- | --- | --- | --- |
| **Patients** | |  | |  |  | | **103** | |  | |
| **General characteristics** | | | | | | | | | | |
| Age. (years); Mean (S.D) | |  | | 22.8 (12.0) | 18.9 (6.81) | | 103 | | 0.052 | |
| Sex; n (%) | | Male | | 16 (33) | 18 (33) | | 34 | | 0.95 | |
|  |  | Female | | 32 (67) | 37 (67) | | 69 | | - | |
| Smoking status; n (%) | | Non-smoker | | 44 (91) | 49 (89) | | 93 | | 0.75 | |
|  |  | Smoker | | 4 (8.5) | 6 (11) | | 10 | | - | |
| Treatment duration (month); Mean (S.D) | |  | | 22.9 (7.84) | 27.7 (9.78) | | 103 | | **<0.01** | |
| Retention duration (month); Mean (S.D) | |  | | 7.48 (5.54) | 20.9 (19.6) | | 103 | | **<0.001** | |
| **Orthodontic characteristics** | | | | | | | | | | |
| Crossbite; n (%) | |  | | 9 (19) | 1 (1.8) | | 10 | | **<0.01** | |
| Overbite (%); Mean (S.D) | |  | | 37.3 (28.7) | 63.8 (28.9) | | 103 | | **<0.001** | |
| Overjet (mm); Mean (S.D) | |  | | 2.99 (2.65) | 3.56 (2.69) | | 103 | | 0.28 | |
| Angle classification. n (%) | | Class I | | 19 (40) | 10 (18) | | 29 | | **<0.001** | |
|  |  | Class II | | 23 (48) | 45 (82) | | 68 | | - | |
|  |  | Class III | | 6 (12) | 0 (0) | | 6 | | - | |
| Arch expansion; n (%) | | Yes | | 9 (20) | 5 (9.4) | | 14 | | 0.15 | |
|  | | No | | 37 (80) | 48 (91) | | 85 | |  | |
| **Cephalometric characteristics** | | | | | | | | | | |
| FMA (°); Mean (S.D) |  | | 24.8 (6.01) | | | 23.3 (5.30) | | 102 | | 0.2 |
| ANB (°); Mean (S.D) |  | | 3.77 (2.19) | | | 4.09 (2.26) | | 102 | | 0.46 |
| I/F at baseline; Mean (S.D) |  | | 113 (7.80) | | | 109 (8.57) | | 101 | | **0.013** |
| IMPA at baseline (°); Mean (S.D) |  | | 93.3 (8.49) | | | 94.3 (8.20) | | 102 | | 0.55 |
| W (mm); Mean (S.D) |  | | 6.81 (1.37) | | | 7.77 (1.48) | | 91 | | **<0.01** |
| H (mm); Mean (S.D) |  | | 20.5 (2.04) | | | 19.1 (2.73) | | 91 | | **<0.01** |
| I/F at T1 (°); Median  [Q25-75] |  | | 113  [109; 115] | | | 106  [106; 106] | | 39 | | 0.21 |
| IMPA after OT (°); Median  [Q25-75] |  | | 93.5  [90.0; 99.0] | | | 100  [100; 100] | | 39 | | 0.29 |
| **Gingival health status** | | | | | | | | | | |
| Gingival inflammation (%); Mean (S.D) |  | | 56.7 (31.8) | | | 24.6 (23.0) | | 103 | | **<0.001** |
| Oral hygiene (%) | Good | | 44.7 | | | 50 | | 103 | | 0.74 |
|  | Poor | | 55.3 | | | 50 | | 103 | | _ |
| Traumatic tooth brushing; n (%) | Mild | | 46.8 | | | 26 | | 103 | | **0.04** |
|  | Moderate | | 53.2 | | | 40 | |  | |  |
| **Periodontal characteristics** | | | | | | | | | | |
| **Teeth** |  | |  | | |  | | **2030** | |  |
| Gingival phenotype; n (%) | Thick and flat | | 418 (45) | | | 529 (48) | | 947 | | 0.11 |
|  | Thin and scalloped | | 516 (55) | | | 567 (52) | | 1083 | | - |
| Gingival recession at baseline; n (%) | 0 | | 912 (98) | | | 1084 (99) | | 1996 | | 0.085 |
|  | ≤1 mm | | 12 (1.3) | | | 6 (0.55) | | 18 | | - |
|  | >1 mm | | 10 (1.1) | | | 6 (0.55) | | 16 | | - |

S.D: standard deviation; OT: orthodontic treatment;

**Supplemental material 3:**

| **Tooth** | **Baseline** | **After active orthodontic treatment** | **Less than 6 months of retention** | **6 to 12 months of retention** | **12 to 24 months of retention** | **More than 24 months of retention** |
| --- | --- | --- | --- | --- | --- | --- |
| **11** | 1 (1%) | 1 (1%) | 0 (0%) | 1 (3.1%) | 0 (0%) | 1 (6.2%) |
| **12** | 1 (1%) | 0 (0%) | 1 (3.3%) | 1 (3.1%) | 0 (0%) | 0 (0%) |
| **13** | 2 (1.9%) | 2 (2%) | 0 (0%) | 2 (6.2%) | 1 (2.7%) | 2 (12.5%) |
| **14** | 2 (2%) | 3 (3.1%) | 2 (7.1%) | 1 (3.1%) | 2 (5.4%) | 1 (6.2%) |
| **15** | 1 (1%) | 0 (0%) | 0 (0%) | 1 (3.1%) | 0 (0%) | 0 (0%) |
| **21** | 4 (3.9%) | 2 (2%) | 0 (0%) | 2 (6.2%) | 0 (0%) | 1 (6.2%) |
| **22** | 1 (1%) | 1 (1%) | 1 (3.4%) | 1 (3.1%) | 1 (2.8%) | 0 (0%) |
| **23** | 3 (2.9%) | 4 (4%) | 1 (3.2%) | 2 (6.2%) | 1 (2.7%) | 2 (12.5%) |
| **24** | 4 (4%) | 3 (3.1%) | 2 (7.1%) | 0 (0%) | 3 (8.1%) | 1 (6.2%) |
| **25** | 1 (1%) | 0 (0%) | 0 (0%) | 0 (0%) | 1 (2.7%) | 0 (0%) |
| **31** | 1 (1%) | 3 (3%) | 2 (6.5%) | 2 (6.2%) | 2 (5.4%) | 0 (0%) |
| **32** | 1 (1%) | 1 (1%) | 1 (3.2%) | 0 (0%) | 0 (0%) | 1 (6.2%) |
| **33** | 0 (0%) | 1 (1%) | 0 (0%) | 1 (3.1%) | 0 (0%) | 0 (0%) |
| **34** | 4 (4%) | 6 (6.2%) | 2 (7.4%) | 1 (3.1%) | 3 (8.1%) | 1 (6.2%) |
| **35** | 1 (1%) | 2 (2.1%) | 1 (3.3%) | 1 (3.2%) | 1 (2.9%) | 1 (6.2%) |
| **41** | 1 (1%) | 4 (4%) | 3 (9.7%) | 2 (6.2%) | 5 (13.5%) | 2 (12.5%) |
| **42** | 1 (1%) | 1 (1%) | 0 (0%) | 0 (0%) | 1 (2.7%) | 1 (6.2%) |
| **43** | 0 (0%) | 0 (0%) | 0 (0%) | 1 (3.1%) | 1 (2.7%) | 0 (0%) |
| **44** | 3 (3%) | 3 (3.1%) | 0 (0%) | 0 (0%) | 4 (10.8%) | 2 (12.5%) |
| **45** | 2 (2%) | 2 (2%) | 2 (6.7%) | 0 (0%) | 2 (5.6%) | 0 (0%) |

**Supplemental material 5:**

|  | **Hospital (n = 46)** | **Private (n = 54)** | **n** | **P value** |
| --- | --- | --- | --- | --- |
|  | Mean (S.D) | |  |  |
| **Satisfaction questionnaire (6-point Likert scale)** |  | |  |  |
| Dental alignment | 5.76 (0.639) | 5.89 (0.317) | 100 | 0.22 |
| Smile | 5.59 (0.580) | 5.48 (0.720) | 100 | 0.42 |
| Facial profile | 4.61 (1.37) | 4.07 (1.48) | 100 | 0.064 |
| Easy of brushing | 4.63 (1.62) | 4.85 (1.16) | 100 | 0.44 |
| Mastication | 4.52 (1.63) | 4.43 (1.38) | 100 | 0.75 |
| Speech | 3.87 (1.88) | 3.91 (1.52) | 99 | 0.91 |
| Lip profile | 4.30 (1.90) | 4.30 (1.45) | 100 | 0.98 |
| Self-esteem | 5.17 (1.10) | 4.91 (1.42) | 100 | 0.29 |
| **Overall satisfaction (0 to 10 scale)** |  |  |  |  |
| Global score | 9.02 (1.02) | 8.96 (1.06) | 100 | 0.78 |

S.D: standard deviation
